# An autopsy case of pulmonary arterial hypertension in an elderly patient with multimorbidity: a case report

**DOI:** 10.1093/ehjcr/ytab527

**Published:** 2021-12-28

**Authors:** Masashi Yokose, Takashi Tomoe, Takehiko Yamaguchi, Takanori Yasu

**Affiliations:** 1 Department of Cardiovascular Medicine and Nephrology, Dokkyo Medical University Nikko Medical Center, 632 Takatoku, Nikko, Tochigi 321-2593, Japan; 2 Department of Pathology, Dokkyo Medical University Nikko Medical Center, 632 Takatoku, Nikko, Tochigi 321-2593, Japan

**Keywords:** Case report, Elderly patients, Multimorbidity, Pulmonary arterial hypertension

## Abstract

**Background:**

There is an increasing number of elderly patients with pulmonary arterial hypertension (PAH), and their characteristics differ from those of young or middle-aged patients with this condition.

**Case summary:**

A 73-year-old woman with a history of myocardial infarction and cardiovascular risk factors was admitted to the hospital with 2-week exertional dyspnoea. Her initial diagnosis was heart failure with preserved ejection fraction, but the symptoms persisted despite receiving treatment with diuretics. Additional tests showed a significant decrease in diffusing capacity of carbon monoxide and findings suggestive of severe pulmonary hypertension (PH). Contrast-enhanced computed tomography of the chest, and pulmonary angiography, showed no narrowing or obstruction of the pulmonary arteries. Right heart catheterization revealed haemodynamic data implying pre-capillary PH. Her condition gradually deteriorated to World Health Organization functional class IV, and sequential combination therapy with tadalafil, macitentan, and selexipag was initiated with a PAH diagnosis; however, she died 1 month later. Pathological findings in autopsy were consistent with PAH, and some parts of the lungs revealed the presence of obstructive and interstitial lung disease.

**Discussion:**

The majority of elderly patients with PAH might have multimorbidity. However, there is no specific treatment strategy. It is associated with diagnostic delay and worse prognosis; therefore, early suspicion and comprehensive tests, including right heart catheterization, are essential for better management.

Learning points

- The number of elderly patients with pulmonary arterial hypertension is increasing. They have multimorbidity and worse prognosis compared with younger patients.
- Pulmonary arterial hypertension in elderly patients might deteriorate rapidly and its diagnosis is challenging; therefore, a high index of suspicion and early comprehensive tests are essential for better management.

## Introduction

Pulmonary arterial hypertension (PAH) usually occurs in young adult women.^1^^,^^2^ Diagnosis requires comprehensive investigation to identify classification, severity, and aetiology. The current treatment strategy is based on disease severity; in severe cases, guidelines recommend specific drug therapy.^3^ Recently, the number of PAH in elderly patients has been increasing.^1^ Herein, we report an autopsy case of PAH in an elderly patient with multimorbidity.

## Timeline

**Table:** 

Day 1	Patient was admitted with a history of 2-week exertional dyspnoea [World Health Organization functional classification (WHO-FC) II]. Tricuspid regurgitation pressure gradient (TRPG) was 54 mmHg on trans-thoracic echocardiogram (TTE).
Day 19	Follow-up TTE showed worsened TRPG (81 mmHg).
Day 36	Right heart catheterization revealed a mean pulmonary arterial pressure of 58 mmHg, pulmonary vascular resistance of 20 Wood units, and normal pulmonary arterial wedge pressure.
Day 56	The patient experienced presyncope and significant hypotension during rehabilitation (WHO-FC IV).
Day 60–90	The patient was treated with sequential triple combination therapy; however, the condition deteriorated.
Day 94	The patient died.

## Case presentation

A 73-year-old obese woman (body mass index, 29.2 kg/m^2^) presented with 2-week exertional dyspnoea. Medical history included inferior myocardial infarction treated with percutaneous coronary intervention in the proximal right coronary artery 17 years ago, dyslipidaemia, hypertension, type 2 diabetes mellitus (DM), and stage 3 chronic kidney disease (CKD); there was no relevant family history. She had a 72-pack-year smoking history until age 56. Medications included aspirin, carvedilol, rosuvastatin, losartan, nifedipine, isosorbide mononitrate, glimepiride, metformin, empagliflozin, and alogliptin.

**Figure 1:**
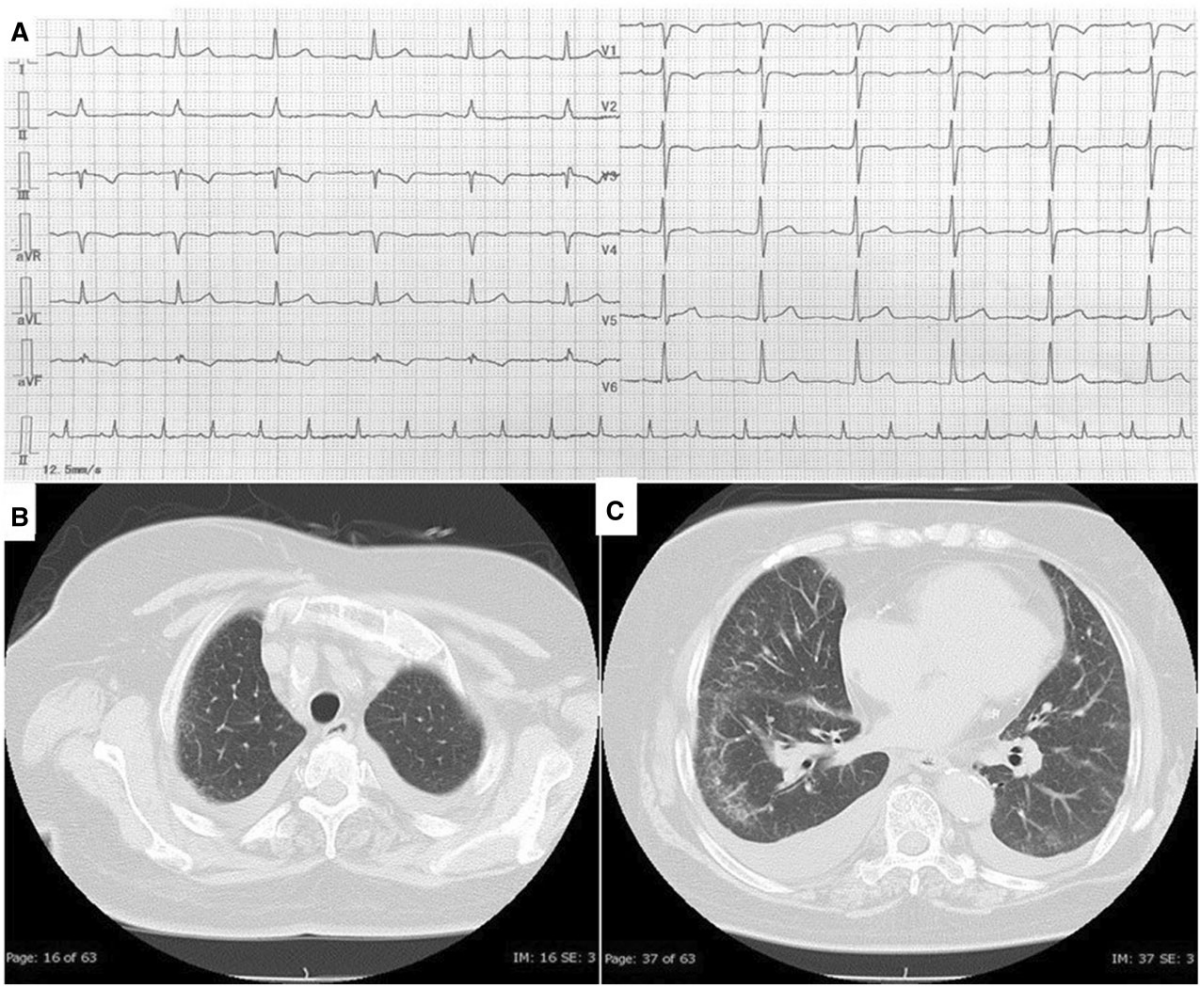
Electrocardiography showed sinus rhythm and inverted T waves in leads II, III, aVF, and V_1, 2_ (*A*). Chest computed tomography without contrast revealed bilateral pleural effusion and right-dominant subpleural ground-glass opacity from the apex (*B*) to base (*C*).

On physical examination, the patient appeared healthy: her heart rate was 77 b.p.m.; blood pressure, 124/66 mmHg; respiratory rate, 18 breath per minute; and percutaneous oxygen saturation (SpO_2_), 95% on 2 L of oxygen. Cardiopulmonary examinations were normal, and laboratory data revealed elevated brain natriuretic peptide (818 pg/mL). Electrocardiography showed sinus rhythm and inverted T waves in leads II, III, aVF, and V_1, 2_ (*Figure 1A*). Chest radiography revealed cardiomegaly, while chest computed tomography (CT) without contrast showed bilateral pleural effusion and subpleural ground-glass opacity (GGO; *Figure 1B* and *C*).

**Figure 2:**
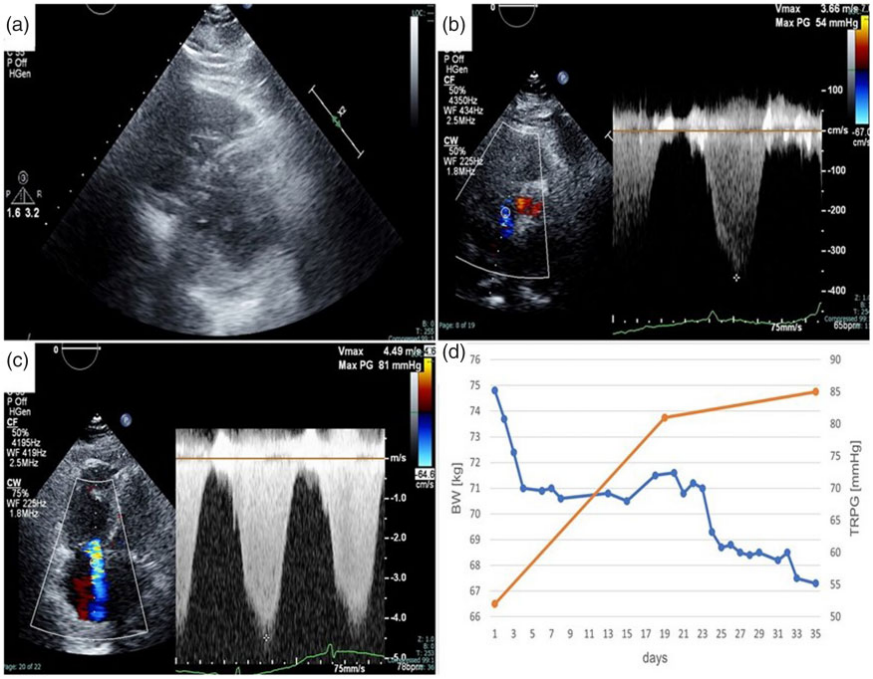
Trans-thoracic echocardiography (TTE) revealed right ventricular dilatation with flattening of the interventricular septum (*A*) and increased tricuspid regurgitation pressure gradient (54 mmHg) (*B*). Follow-up TTE on Day 19 revealed worsened tricuspid regurgitation pressure gradient (81 mmHg) (*C*) despite the patient losing body weight due to diuretic therapy (*D*).

Trans-thoracic echocardiography (TTE) revealed a normal sized, hypertrophic (wall thickness, 12 mm) left ventricle (LV) with normal ejection fraction (65%), cardiac output (4.15 L/min), and cardiac index (2.28 L/min/m^2^); and signs of diastolic dysfunction (mitral E/A, 0.70; mitral E velocity deceleration time, 285 ms; average E/é, 18.9). The right ventricle (RV) was hypertrophic and dilated, with a flattened interventricular septum and increased tricuspid regurgitation pressure gradient (TRPG, 54 mmHg; *Figure 2A* and *B*). The estimated right atrial pressure (RAP) was 8 mmHg, and the inter-arterial septum remained intact. The patient was diagnosed with heart failure with preserved ejection fraction, and furosemide (10 mg/day) was administered.

She lost 4 kg of weight within 4 days; however, her symptoms sustained. Arterial blood gas analysis while breathing ambient air revealed a PaCO_2_ of 37 mmHg, PaO_2_ of 61.4 mmHg, SaO_2_ of 91.7%, and alveolar-arterial oxygen gradient of 42.7 mmHg. The pulmonary function test (PFT) showed a reduced forced vital capacity (FVC) of 1.82 L (73.6% of predicted), reduced forced expiratory volume in 1 s (FEV1) of 1.39 L (72.7% of predicted), mildly reduced FEV1/FVC of 76.3%, and markedly reduced diffusing capacity of carbon monoxide (DLCO) of 5.76 mL/min/mmHg (30.4% of predicted). The 6 min walking test revealed reduced distance (88 m) and temporarily declined SpO_2_ (80%).

**Figure 3:**
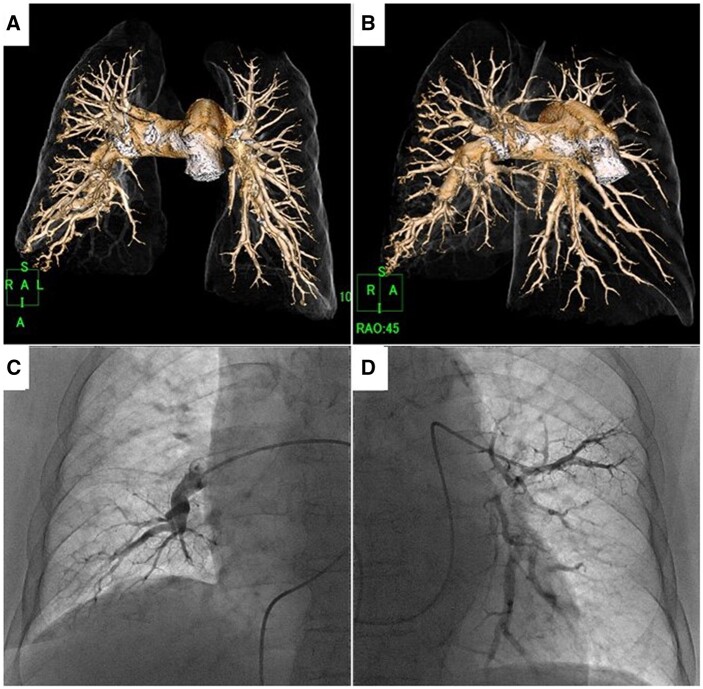
Contrast-enhanced computed tomography of the chest with three-dimensional reconstruction detected no pulmonary embolism (*A*; frontal view, *B*; right anterior oblique view). Pulmonary angiography showed no narrowing or obstruction of the pulmonary artery (*C*; right middle and lower lobe segment in frontal view, *D*; left lingual and lower lobe segment in frontal view).

On the 19th day of admission, follow-up TTE showed a markedly hypokinetic and enlarged RV (40 mm), with decreased systolic function (tricuspid annular plane systolic excursion, 13.4 mm) and worsened TRPG (81 mmHg; *Figure 2C* and *D*); estimated RAP remained 8 mmHg. Peak tricuspid regurgitation velocity was 4.5 m/s, consistent with a high probability of pulmonary hypertension (PH).^3^ Contrast-enhanced chest CT detected no pulmonary embolism (*Figure 3A* and *B*). Right heart catheterization (RHC) revealed elevated mean pulmonary arterial pressure (58 mmHg) and pulmonary vascular resistance (20 Wood units), normal mean pulmonary arterial wedge pressure (13 mmHg), and cardiac output/cardiac index (3.2 L/min and 1.9 L/min/m^2^), respectively. Vasoreactivity testing and fluid challenge were not performed. There was no significant increase in oxygen saturation between arterial and venous blood samples. Pulmonary angiography demonstrated no narrowing or obstruction (*Figure 3C* and *D*). Coronary angiography was almost unremarkable except for 75% stenosis in the distal left circumflex artery. She was diagnosed with pre-capillary PH,^1^^,^^2^ and PAH was highly suspected. Additional tests for serum anti-human immunodeficiency virus antibody and autoantibodies associated with connective tissue diseases were all negative.

**Figure 4:**
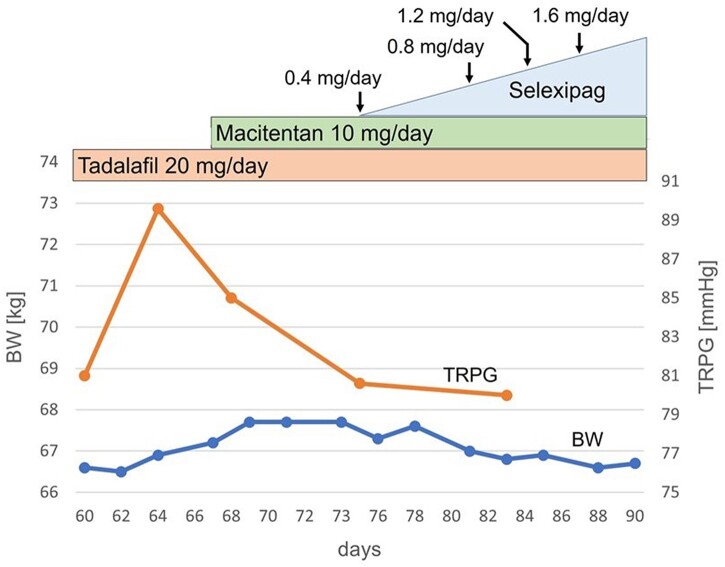
Association between sequential combination therapy, tricuspid regurgitation pressure gradient, and body weight. BW, body weight; TRPG, tricuspid regurgitation pressure gradient.

She was unable to undergo a ventilation/perfusion lung scan to exclude chronic thrombo-embolic PH (CTEPH) due to presyncope, progressive dyspnoea at rest, and significant hypotension. She was diagnosed as World Health Organization functional class (WHO-FC) IV and treated with sequential combination therapy—including tadalafil (20 mg/day, phosphodiesterase type 5 inhibitor) and macitentan (10 mg/day, endothelin receptor antagonist) after 2 weeks—which was ineffective (*Figure 4* and Supplementary material online, *Table S1*). The third medication—selexipag (oral prostacyclin receptor agonist)—was added, its dose gradually increased; however, the patient’s respiratory status rapidly deteriorated on the 90th day of admission. She refused intubation and died 4 days later.

**Figure 5:**
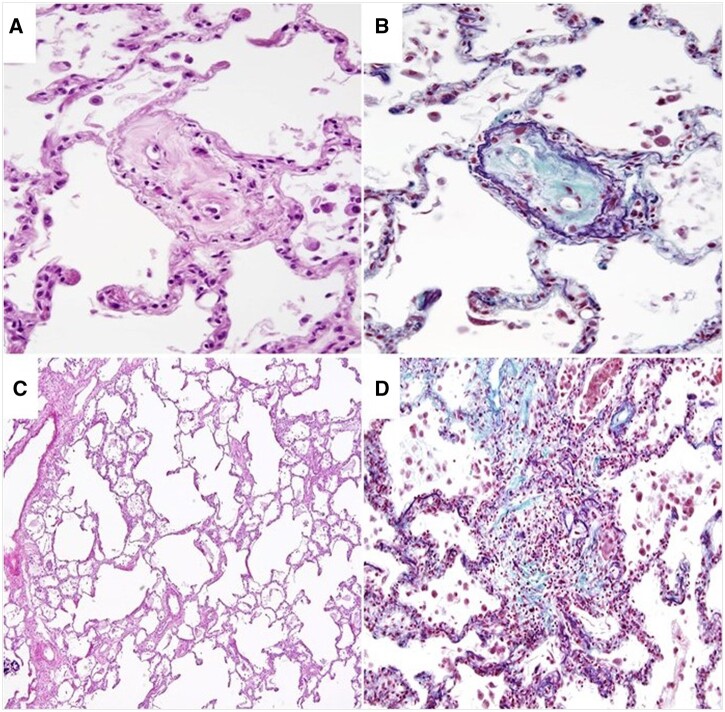
Autopsy findings (*A* and *B*). Smaller pulmonary arterioles exhibit cellular intimal proliferation resulting in prominent luminal stenosis (*A*: Haematoxylin and eosin staining, 400×). (*B*) Masson Trichrome staining, 400×. (*C*) Emphysematous pulmonary parenchyma and intra-alveolar aggregates of macrophages (haematoxylin and eosin staining, 100 ×). (*D*) Mildly thickened alveolar septa and focal fibrosis with chronic inflammatory cell infiltration (Masson Trichrome staining, 200×).

An autopsy after 30 h (*Figure 5A–D*) revealed medial thickening and prominent luminal stenosis due to cellular intimal proliferation of pulmonary arterioles, especially the smaller ones. Furthermore, there were emphysematous parenchymal changes, thickened alveolar septa, and focal fibrosis, consistent with organizing pneumonia. Many lymphocytes and macrophages infiltrated alveoli. The enlarged heart exhibited focal fibrosis and myocardial fascicular arrangement of the LV posterior wall, consistent with old myocardial infarction. The RV wall was hypertrophic and mildly dilated. Focal amyloid deposits at hyalinized islets of Langerhans in the pancreas, renal arteriosclerosis, and arteriolosclerosis, and scattered globally sclerotic glomeruli indicated DM.

## Discussion

Currently, there are only a few case reports which confirmed rapidly progressive PAH in elderly patients histologically. Guidelines do not describe PAH in elderly individuals with multimorbidity;^3^ therefore, diagnosis and management were based on the procedure for young or middle-aged patients.^1^^,^^3^^,^^4^ If PH is likely, diagnostic evaluations such as PFT should be performed to exclude Group 2 or 3 PH. Guidelines then recommend a ventilation/perfusion scan to evaluate CTEPH; if CTEPH is unlikely, RHC—and additional studies including magnetic resonance imaging^5^ and specific serology testing—are considered to classify PAH. In our case, pulmonary findings on imaging and histology may suggest Group 3 PH; however, her PFT showed a mild restrictive pattern with disproportionately reduced DLCO, which could not explain severe PH. Additionally, GGO is present in more than one-third of PAH patients;^6^ thus, we concluded the patient had Group 1 PH with concomitant lung disease, rather than PH due to lung disease (Group 3 PH).

Hjalmarsson *et al*.^7^ previously reported about 20% of elderly patients >65 years old with idiopathic PAH have at least four comorbidities, including hypertension, DM, ischaemic stroke, ischaemic heart disease, atrial fibrillation, obesity, and CKD. Hoper *et al*.^5^ claimed they camouflage non-specific symptoms of PAH—such as fatigue—which leads to diagnostic delay. Our patient had most of these comorbidities, which may have contributed to delayed suspicion and performance of specific tests for PAH.

While specific drug therapy for PAH comprises three different signalling pathways, elderly patients often undergo less aggressive treatment.^8^ Some studies have demonstrated a worse prognosis in elderly patients than in young or middle-aged patients.^8^^,^^9^ According to the UK/Ireland registry, 1-, 2-, 3-, and 5-year survival rates in patients >50 years old were 90%, 75.5%, 57.1%, and 43.7%, respectively.^10^ We did not use the continuous intravenous prostacyclin analogues which is the 1st line therapy of WHO-FC IV PAH,^3^ as the patient refused; consequently, symptoms rapidly progressed within 3 months. Clinicians must recognize the condition of elderly patients might deteriorate quickly; therefore, early suspicion and comprehensive diagnostic tests are critical for better management.

## Conclusions

Pulmonary arterial hypertension is seen in young or middle-aged adults, as well as elderly people. Its diagnosis and management are challenging; therefore, a high index of suspicion and early comprehensive tests are essential for better patient outcomes.

## Lead author biography

**Figure:**
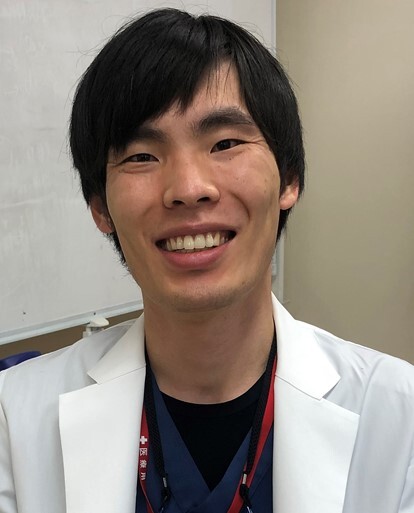


Dr Masashi Yokose is a PGY5 doctor enrolling in the internal medicine fellowships at Dokkyo Medical University Hospital in Japan. He worked at Dokkyo Medical University Nikko Medical Center to receive training in cardiology and nephrology in 2020. His main interests are general internal medicine and critical care.

## Supplementary material

Supplementary material is available at *European Heart Journal - Case Reports* online.

## Supplementary Material

ytab527_Supplementary_Data

## References

[1] Sitbon O , Howard L. Management of pulmonary arterial hypertension in patients aged over 65 years. Eur Heart J Suppl 2019;21:K29–K36. 31857798 10.1093/eurheartj/suz206 PMC6915056

[2] Pugh ME , Sivarajan L, Wang L, Robbins IM, Newman JH, Hemnes AR. Causes of pulmonary hypertension in the elderly. Chest 2014;146:159–166. 24480915 10.1378/chest.13-1900 PMC4077408

[3] Galie N , Humbert M, Vachiery JL, Gibbs S, Lang I, Torbicki A et al; ESC Scientific Document Group. 2015 ESC/ERS Guidelines for the diagnosis and treatment of pulmonary hypertension: the Joint Task Force for the Diagnosis and Treatment of Pulmonary Hypertension of the European Society of Cardiology (ESC) and the European Respiratory Society (ERS): endorsed by: Association for European Paediatric and Congenital Cardiology (AEPC), International Society for Heart and Lung Transplantation (ISHLT). Eur Heart J 2016;37:67–119. 26320113 10.1093/eurheartj/ehv317

[4] Frost A , Badesch D, Gibbs JSR, Gopalan D, Khanna D, Manes A et al Diagnosis of pulmonary hypertension. Eur Respir J 2019;53:1801904. 30545972 10.1183/13993003.01904-2018 PMC6351333

[5] Aryal SR , Sharifov OF, Lloyd SG. Emerging role of cardiovascular magnetic resonance imaging in the management of pulmonary hypertension. Eur Respir Rev 2020;29:190138. 32620585 10.1183/16000617.0138-2019 PMC9488921

[6] Rajaram S , Swift AJ, Condliffe R, Johns C, Elliot CA, Hill C et al CT features of pulmonary arterial hypertension and its major subtypes: a systematic CT evaluation of 292 patients from the ASPIRE Registry. Thorax 2015;70:382–387. 25523307 10.1136/thoraxjnl-2014-206088 PMC4392204

[7] Hoeper MM , Simon R Gibbs J. The changing landscape of pulmonary arterial hypertension and implications for patient care. Eur Respir Rev 2014;23:450–457. 25445943 10.1183/09059180.00007814 PMC9487398

[8] Hjalmarsson C , Rådegran G, Kylhammar D, Rundqvist B, Multing J, Nisell MD et al Impact of age and comorbidity on risk stratification in idiopathic pulmonary arterial hypertension. Eur Respir J 2018;51:1702310. 29622568 10.1183/13993003.02310-2017

[9] Hoeper MM , Huscher D, Ghofrani HA, Delcroix M, Distler O, Schweiger C et al Elderly patients diagnosed with idiopathic pulmonary arterial hypertension: results from the COMPERA registry. Int J Cardiol 2013;168:871–880. 23164592 10.1016/j.ijcard.2012.10.026

[10] Ling Y , Johnson MK, Kiely DG, Condliffe R, Elliot CA, Gibbs JS et al Changing demographics, epidemiology, and survival of incident pulmonary arterial hypertension: results from the pulmonary hypertension registry of the United Kingdom and Ireland. Am J Respir Crit Care Med 2012;186:790–796. 22798320 10.1164/rccm.201203-0383OC

