# Supplementary material for: An autopsy case of pulmonary arterial hypertension in an elderly patient with multimorbidity: a case report
Source: Eur Heart J Case Rep. 2021 Dec 28;6(1):ytab527. doi: 10.1093/ehjcr/ytab527 (PMC8753136; doi:10.1093/ehjcr/ytab527)
Supplement: ytab527_Supplementary_Data [file ytab527_supplementary_data.docx]

**Table S1.** Serial changes in echocardiographic parameters since admission.

|  | RV dimension  [mm] | TRPG  [mmHg] | TAPSE  [mm] | Cardiac index  [L/min/m^2^] |
| --- | --- | --- | --- | --- |
| Day 1 |  | 54.0 |  | 2.28 |
| Day 19 | 40.4 | 81.0 | 13.4 |  |
| Day 35 |  | 85.0 | 11.3 |  |
| Day 60* | 43.2 | 81.0 | 12.2 | 1.5 |
| Day 64 |  | 89.6 |  |  |
| Day 68 |  | 85.0 | 11.7 | 2.8 |
| Day 75 |  | 80.6 |  |  |
| Day 83 | 43.2 | 80.0 | 14.8 |  |

Blank space indicates that the parameter was not measured. *The first day of sequential combination therapy. RV, right ventricle; TRPG, tricuspid regurgitation pressure gradient; TAPSE, tricuspid annular plane systolic excursion.
